# PDL1 expression on monocytes is associated with plasma cytokines in Tuberculosis and HIV

**DOI:** 10.1371/journal.pone.0258122

**Published:** 2021-10-01

**Authors:** Wegene Tamene, Meseret Abebe, Liya Wassie, Helina Mollalign, Katrin Bauer, Amha Kebede, Vincent C. Marconi, Rawleigh Howe, Ulrich Sack

**Affiliations:** 1 HIV and TB Research Directorate, Ethiopian Public Health Institute (EPHI), Addis Ababa, Ethiopia; 2 Mycobacterial Disease Research Directorate, Armauer Hansen Research Institute (AHRI), Addis Ababa, Ethiopia; 3 Institute of Clinical Immunology, Medical Faculty, University of Leipzig, Leipzig, Germany; 4 School of Medicine and Rollins School of Public Health, Emory University, Atlanta, Georgia, United States of America; Rutgers Biomedical and Health Sciences, UNITED STATES

## Abstract

**Introduction:**

PDL1 and its interaction with PD1 is implicated in immune dysfunction in TB and HIV. The expression of PDL1 on multiple subsets of monocytes as well as their associations with cytokines and microbial products have not been well studied.

**Method:**

HIV (TB-HIV+), TB (TB+HIV-) and TB/HIV co-infected (TB+HIV+) patients as well as apparently healthy controls (TB-HIV-) were recruited. TB and HIV patients were treatment naïve while TB/HIV patients were both ART naïve and experienced but not yet started TB therapy. Monocyte subsets were evaluated for PDL1 expression by flow cytometry; plasma TNFα, IL6, IP10, IFNγ and IL10 were measured by Luminex; and cytokine mRNA from purified monocytes quantitated by qPCR. The association of PDL1 with cytokines, clinical and microbial indices, including HIV viral load, TB smear microscopy and TB urinary lipoarabinomannan (LAM) were assessed.

**Results:**

Monocyte expression of PDL1 was significantly higher in TB, HIV and TB/HIV co-infected patients compared with healthy controls (p = 0.0001), with the highest levels in TB/HIV co-infected patients. The highest expression of PDL1 was on intermediate (CD14+CD16+) monocytes in all participant groups. PDL1 strongly correlated with HIV viral load in TB/HIV while weakly correlated in HIV. PDL1 levels moderately correlated with plasma TNFα, IL6, IP10, IFNγ and IL10 level in TB subjects whereas weakly correlated with TNFα and IP10 in HIV patients. However, cytokine mRNA from purified monocytes showed no association with either plasma cytokines or monocyte PDL1 expression, implying that if cytokines modulate PDL1, they are likely not originating from circulating monocytes themselves. These results underscore the importance of further characterization of multiple monocyte subsets and their phenotypic and functional differences in different disease states.

## Introduction

Tuberculosis (TB) is among the top ten causes of deaths worldwide. According to World Health Organization (WHO), globally there were 10 million new diagnoses of TB with 1.2 million deaths reported in 2019 [1]. In sub-Saharan Africa, the incidence of TB is aggravated by high rates of HIV co-infection, another serious disease, in the region. The possibility of developing TB disease from TB infection is increased from 5–10% of those infected with TB in their entire lifetime [2] to 20–30% within 2 years for individuals with HIV, underscoring the additional impact of HIV on TB disease.

There are many immune system abnormalities reported in HIV as well as TB disease. One of the critical immuno-regulatory pathways effected is the PD1-PDL1 pathway, whereby induced PD1 on T cells and PDL1 on antigen presenting cells can result in T cell dysfunction in chronic infections with persistent antigen exposure [3, 4]. This has been shown in TB and HIV infections, and as well in cancer where extended exposure of cancer neoantigens to cells in a tumor microenvironment results in T cell exhaustion. Importantly, recovery of T cell function and improved disease outcomes can be achieved through therapy with antagonists to PD1 and PDL1, and with other checkpoint inhibitors [5].

In chronic infectious diseases such as TB and HIV, the role of PD1 on T cells, and to a lesser extent the role of PDL1 on dendritic cells has been well-studied [4, 6, 7]. Fewer studies, however, have addressed monocytes or their subsets, particularly in the African context. Moreover, although many animal or *in-vitro* human studies have addressed factors involved in modulating PDL1 expression, there is very little relevant *ex-vivo* data from human subjects. The goals of the present study, therefore, were to define the expression of PDL1 on multiple monocyte subsets in TB, HIV and co-infected patients, and determine the association of PDL1 with indices of microbial load, plasma cytokines and monocyte RNA encoding such cytokines which may play a role in PDL1 modulation *in-vivo*.

## Methods

### Study site and patient population

Newly diagnosed TB (TB+ and HIV-), HIV (TB- and HIV+), TB/HIV co-infected (TB+ and HIV+) patients and apparently health controls (TB- and HIV-), aged 18 and above were recruited from selected health facilities in Addis Ababa, Ethiopia. Patients with a history of previous TB, autoimmune disorders, chronic diseases such as diabetes, or those on corticosteroid medication were excluded. The TB and TB/HIV cases were newly diagnosed TB cases before start of anti-TB treatment. The TB patients’ diagnosis was confirmed bacteriologically in the majority of cases (83.9%) while the rest determined based on clinical and radiological diagnosis according to WHO criteria. Similarly, the TB/HIV subjects were either–bacteriologically confirmed (90.9%) or based on clinical and radiological criteria (9.1%). All HIV patients were treatment naïve while TB/HIV co-infected patients were recruited irrespective of their ART status. Apparently healthy controls were recruited from clients visiting the same health facilities for routine medical checkup or voluntary counseling and testing (VCT) services. The inclusion criteria for apparently healthy controls included all the aforementioned inclusion criteria of the clinical groups as well as no current clinical signs of TB and tested HIV negative. HIV testing was performed at our laboratory as per the National HIV testing algorithm. The study received ethical approval from the National Research Ethics Review Committee of Ethiopia and Ethiopia Public Health Institute review boards prior to participant recruitment. All study participants gave written consent prior to their enrollment.

### Isolation of Peripheral Blood Mononuclear Cells (PBMC)

Approximately, 20 ml of venous blood samples were collected from all participants using heparinized tubes, before participants were initiated on anti-TB treatment (for newly TB diagnosed TB and TB/HIV patients) or ARV for those with HIV. In addition, 4ml of blood was collected in EDTA containing tubes from HIV and TB/HIV co-infected participants for CD4 count and HIV viral load determination. 10 ml of plasma was separated from freshly collected heparinized blood and stored at -80°C in 2 ml aliquot volumes until assayed further. The remaining blood was mixed with equal volumes of RPMI (Sigma) and layered over Ficoll-paque plus (GE) using Leucosep tubes (Greiner). Interface cells were harvested and washed three times with phosphate buffered saline (PBS). Cells were finally resuspended in 1ml of RPMI and counted with trypan blue staining to exclude of non-viable cells. On average, the viability cell count ranged between 95 and 100%. All specimens were treated as infectious material and handled as per the laboratory’s biosafety guidelines based on international standards.

### Cell surface staining for flow cytometry

One million freshly isolated PBMCs were used for cell surface marker staining in two separate 4 cc polystyrene tubes (BD, USA), where one of the tubes contained anti-CD14 PerCP (BD, USA), CD16 APC-H7, PDL1 PE (Biolegend, Germany) and the other tube contained IgG2b isotype control for PDL1 antibody along with the aforementioned fluorochrome conjugated anti-CD14 and anti-CD16. The cells were stained for 20 minutes in the dark at room temperature, washed with FACS buffer (PBS containing 1mM EDTA and 0.1% bovine serum albumin), and the resuspended pellet fixed with 0.5 ml 4% paraformaldehyde for 20 minutes. Finally, cells were washed, re-suspended in FACS buffer and data was acquired using a FACSCanto II cytometer with FACSDiva software (BD, USA), and analyzed using Flowjo 9.4.6 Software (FlowJo, USA). We included an internal quality control consisting of PBMC from a healthy control which were stained, fixed, frozen in replicate aliquots and thawed periodically as a reference to guide appropriate gating. Samples resulting in fewer than 1700 acquired monocytes and those samples with clearly suboptimal staining were excluded from analysis.

### Plasma cytokine analysis

Stored plasma specimens (never been thawed) were used to measure plasma cytokine level using human premixed multi-analyte kit (R&D, Germany) according to the manufacturer’s instructions (R&D, Germany). Frozen plasma specimens were thawed and centrifuged briefly to remove sediment and supernatant transferred to new 1.5 ml Eppendorf tubes. Samples were diluted two-fold with kit buffer and randomly positioned in the reaction plates. Six standard points were included in each run. Both specimens and standards were tested in duplicate. Coefficient of variation (CV) between duplicate sample was assessed for all plasma specimens resulting in a mean CV of 8% (range: 4–10%). Internal luminex assay controls (low and high) were used as a quality control tool. To avoid lot to lot variation, reagents from the same lot were used for all specimens. Standards and samples were acquired on MAGPIX Luminex machine (xMAP Tech, Germany), concentration of cytokines then determined by using xPONANT v4.2 software. The level of cytokines was read as MFI then converted to concentration based on the standard curve for each marker. A panel of ten inflammatory cytokines: IFNγ, IFNα, IL-1α, IL-1β, TNFα, IL-6, IL-10, IL-12p70, IL-17 and IP10 were included in the plasma cytokine analysis. The cytokines included in the study was selected based on their relevance in TB and HIV disease as well as their involvement with PDL1 expression as well as documented on previous reviews [8–10].

### Cytokine mRNA analysis

#### Monocyte purification

Ten million freshly isolated PBMCs were washed with MACS buffer (PBS with Tween 20), resuspended and fractionated using the MACS monocyte isolation kit II (Miltenyi Biotech, Germany) according to the manufacturer’s instruction. The fractionation process was repeated an additional cycle to ensure high purity. Eluted cells were washed, and 350μl of RNA protect (Qiagen, Germany) was added to resuspended pellet and cells were stored at -80°C until use.

#### RNA isolation and cDNA preparation

Monocytes in RNA protect were thawed slowly and washed with sterile PBS and centrifuged at 5000g for 5 minutes. The supernatant was completely aspirated, and cell pellet loosened by gentle flicking. RNA was isolated using the Qiagen RNeasy micro kit (Qiagen, Germany) as per the manufacturer’s instruction. RNA concentration and purity were checked by the Nano Drop 2000 Spectrophotometer (Thermo Scientific, Epsom, UK) and RNA integrity by agarose gel electrophoresis. mRNA was then converted into cDNA using the Omniscript Reverse Transcriptase kit (Qiagen, Germany) in a 20μl final reaction volume. The master mix for the cDNA preparation was as follows: 10x RT buffer (1.0ml), 25x dNTP mix (100nm), 10x RT random primer (1.0ml), RNase inhibitor (100μl), reverse transcriptase 50 U/μL, and nuclease-free water to a total 10μl reagent volume, and 10μl template RNA to final reaction volume of 20μl. Reactions were incubated in an ABI9700 Programmable Thermal Cycler (Applied Biosystems, Foster City, California) for 10 minutes at 25°C followed by 60 minutes at 37°C and 5 minutes at 85°C then cooling to 4°C according to the manufacturer’s instructions.

#### Gene quantification

Gene expression of TNFα and IL10 in fractionated monocytes were quantified using qPCR in Rotor-Gene™ 3000 thermal cycler (Corbett Life Science, Crawley, UK) using the Rotor-gene® SYBR® Green PCR Kit (Qiagen, Crawley, UK). The primer sequences of TNFα and IL10 were as follows: TNFα forward primer (FP) 5’AGCCCATGTTGTAGCAAACC3’ and reverse primer (RP) 5’GCTGGTTATCTCTCAGCTCCA3’, IL10 FP 5’TGAGAACCAAGACCCAGACA3’ and RP 5’TCATGGCTTTGTAGATGCCT3’. Human Ribosomal Protein (HuPO) was used as reference gene to normalize gene expression. HuPO’s primer sequences are FP 5’GCTTCCTGGAGGGTGTCC3’ and RP 5’GGACTCGTTTGTACCCGTTG3’. The PCR reaction with a final volume of 12.5μl consisted of 6.25μl 1x Rotor-gene SYBR Green PCR hot start master mix, 1μM each of forward and reverse primers, 2.5μl cDNA and 4.25μl of RNase free water. The PCR reaction was set as a onetime 15 min initial Taq enzyme activation at 95°C followed by 40 cycles of 5 sec denaturation at 95°C, 10 sec primer annealing at 60°C and 20 sec extension at 72°C. In each run the above reaction mix without cDNA was used as a negative control. Each sample was run in duplicate, and average of the duplicate (average Ct) was used for analysis. Data was analyzed using the relative quantification method as described by Livak and Schmittgen [11].

### Microbial indices and CD4 count

Along with heparinized blood, sputum and urine samples were collected from TB and TB/HIV patients and stored at -20°C using sterile containers for bacteriological and lipoarabinomannan (LAM) analysis, respectively. In addition, EDTA blood was collected for CD4 count and HIV viral load determination from HIV and TB/HIV participants. Sputum culture and acid-fast bacilli (AFB) smear examination were performed at the National TB reference Laboratory, EPHI, according to the laboratory’s standard operating procedure (SOP). Urine specimens, collected at the time of participant recruitment, were thawed, centrifuged and then the supernatants were used for LAM testing using Determine LAM Ag lateral flow assay as per the manufacturer’s instruction (Alere, USA). The assay was interpreted as a semi-quantitative result based on the visual chart provided by the manufacturer. CD4 count was performed on FACSCalibur (BD) while HIV virus load (VL) was determined using Cobas Amplipre/Taqman according to SOPs of National HIV Laboratory, EPHI.

### Data analysis

All statistical analyses were performed on GraphPad Prism 6.0 (GraphPad software, La Jolla California USA). Comparison between groups was made using non-parametric Kruskal-Wallis followed by Dunn’s multiple comparison test. Data on group comparison was presented as median with interquartile ranges. Correlation between variables was assessed using the non-parametric Spearman correlation test. The findings of correlation are presented in correlation coefficient (r) along with p-values. Whenever correlations presented in qualitative terms such as weak, moderate and strong correlation, a paper by Mukaka 2012 was used as a source document [12]. Fold-change in gene expression was calculated using Microsoft Excel as 2^-delta delta Ct (2^-ΔΔCt). Results were considered statistically significant with p-values less than 0.05.

## Results

### Participants’ characteristics

Demographic characteristics of study participants are presented in Table 1. All TB patients were bacteriologically confirmed (n = 34) while the participants with TB/HIV co-infection (n = 12) were either bacteriologically confirmed or diagnosed based on clinical and radiological criteria. All the participants with HIV (n = 35) were ART naive. The median body mass indices (BMI) were 18.3, 20.6 and 19.6 for participants with TB, HIV and TB/HIV, respectively. As expected, the participants with TB/HIV had higher mean VL than the HIV cases (1,821,679 [20–14,881,766] and 297,877 [20–3,506,397] copies/ml, respectively).

**Table 1:** Basic demographic and clinical characteristics of study participants.

Group	*N*	Age	Sex	ART status		Viral load
		*Median*	*Male*: *Female*	*Naïve*	*on ART*	*Mean copies/ml (range)*
**Healthy Controls**	39	28	1.5			
**TB**	34	30	2.6			
**HIV**	35	33	0.7	35		297,877 (LDL-3,506,397)
**TB/HIV**	12	41	0.9	7	5	1,821,679 (LDL- 14,881,766) ^a^

Mean & range of viral load copies/ml 2,976,946 (LDL– 14,881,766) for ART naïve, and 510,073 (LDL– 1,995,707) for patients on ART; LDL: lower detectable limit (20 copies/ml).

### Defining monocyte subsets

**Fig 1:**
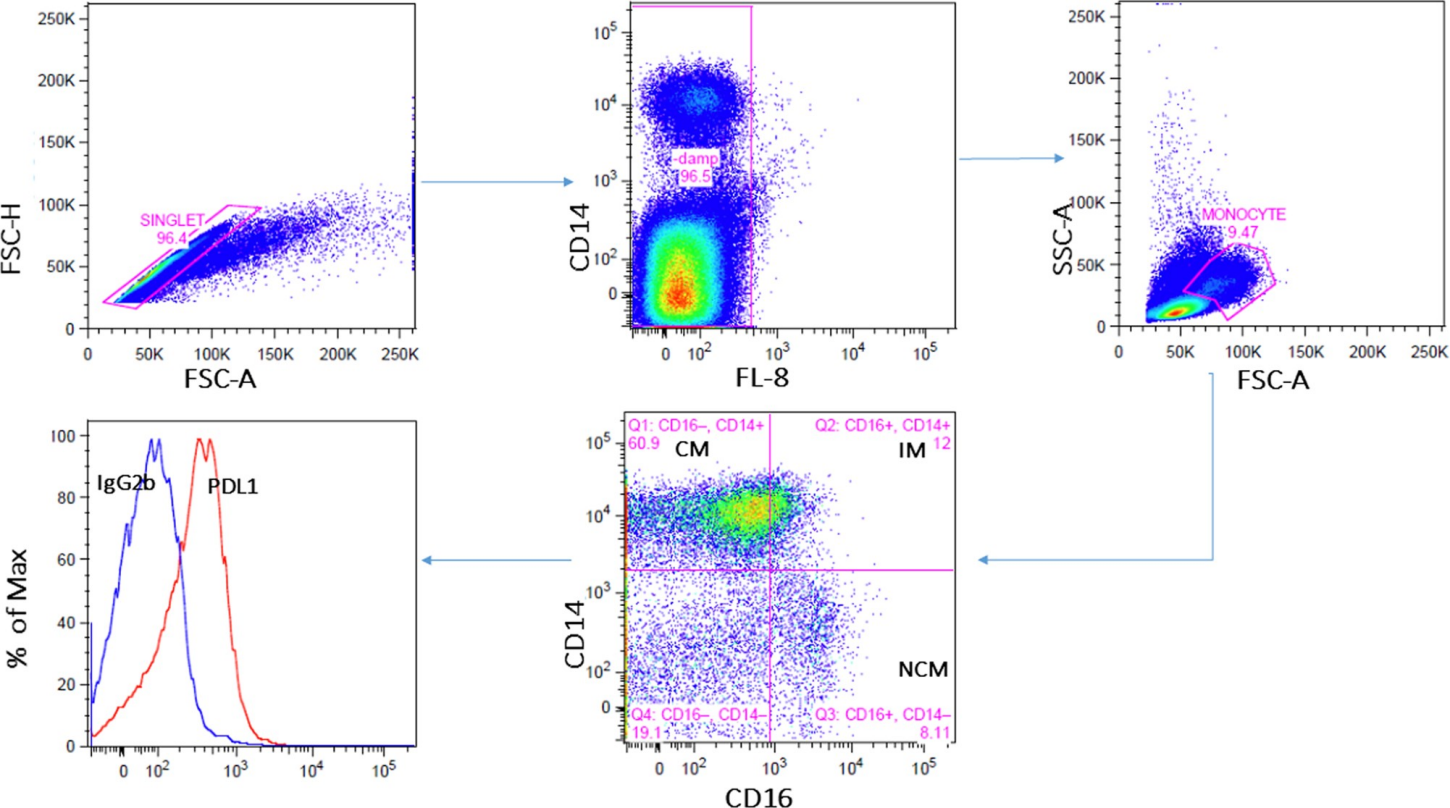
Gating strategy for monocyte, their subsets and marker antibody-isotype difference. Doublets were excluded using FSC-H and FSC-A followed by exclusion of non-specific staining cells defined by using the FL8 detector as a dump channel (see text). Total monocytes were enriched based on a FSC and SSC gate and then classified into classical, intermediate and non-classical monocyte subsets based on the expression of CD14 and CD16. Specific PDL1 intensity of expression was determined by subtracting the MFI from control tube containing CD14, CD16 and an IgG2b isotype control.

Following flow cytometric acquisition, monocytes were gated by first excluding doublets by light scatter properties. Fluorescent positive cells detected in the FL8 channel were assumed to be non-specifically stained (since no FL8 specific fluorescent marker was used) and gated out. Monocyte enriched populations were selected based on forward and perpendicular light scatter. Light scatter gated monocytes were defined by the expression of CD14 and CD16, and further delineated into CD14+CD16- (classical), CD14+CD16+ (intermediate), and CD14-CD16+ (non-classical) subsets as illustrated in Fig 1. Finally, the net median fluorescence intensity (nMFI) of PDL1 on gated monocytes (see Fig 1) was computed by subtracting the median fluorescence intensity (MFI) of a separate tube containing CD14, CD16 and an appropriate isotype (IgG2b) control antibody. The mean total numbers of acquired events were 293,687 (standard deviation 166,637) and the average total monocytes count was 19,463 (standard deviation 12,897).

### TB, HIV and TB/HIV Co-infected participants had high PDL1 expression on monocytes subsets

**Fig 2:**
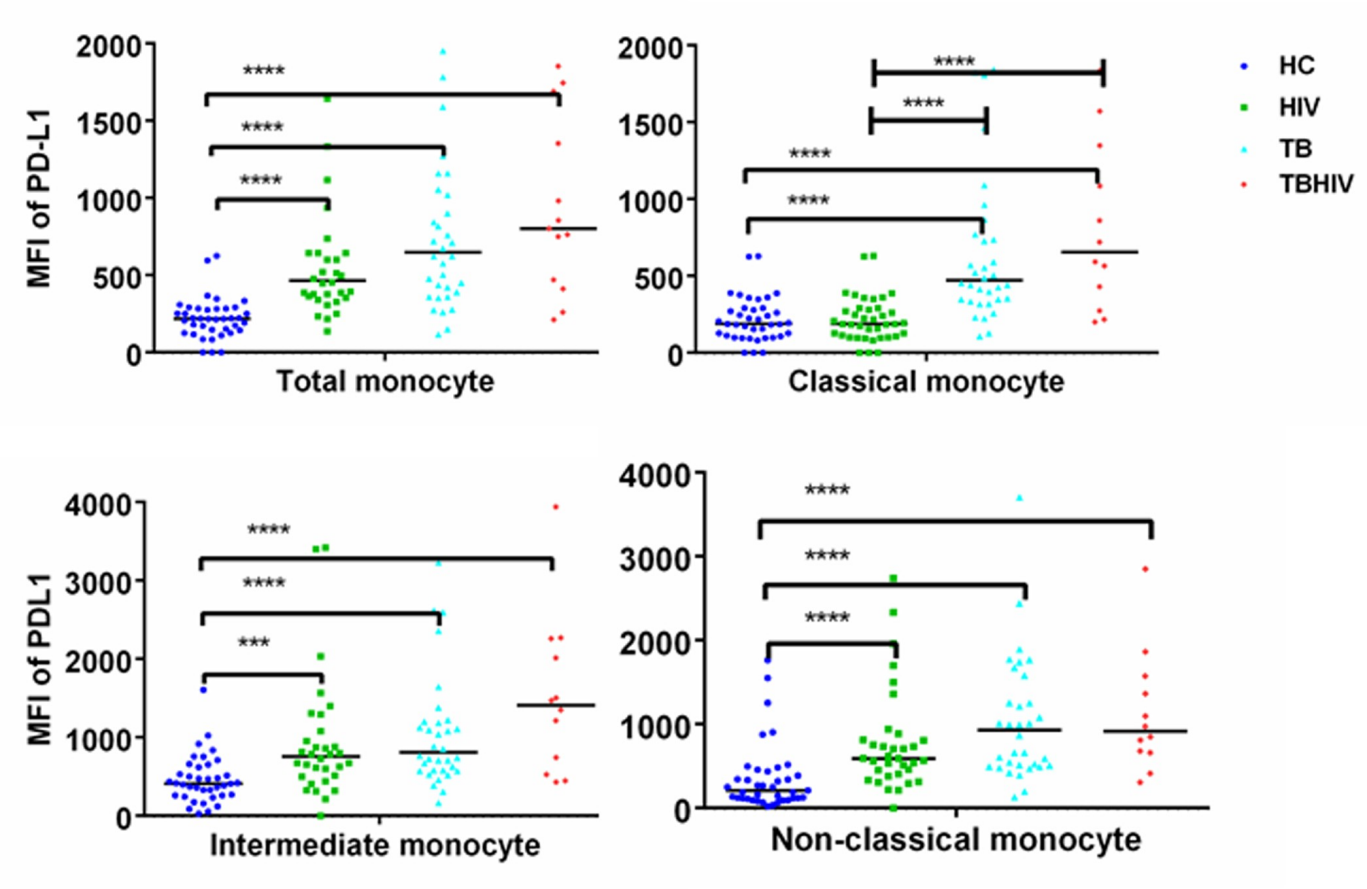
Expression of PDL1 as presented in participants group on total monocytes and monocyte subsets. The graphs represent the expression of PDL1 in participants group on total monocytes (a), CM (b), IM (c) and NCM (d). The expression is presented as net MFI computed after subtraction of isotype control values (see materials and methods) Dot plots are shown indicating median, and interquartile range. The Asterisks represents P-values of: *0.05, **0.01 ***0.001 &****0.0001. Non-parametric Kruskal-Wallis test followed by Dunn’s multiple comparison was used.

PDL1 expression was evaluated in different clinical groups, and was found to be three-fold higher in participants with HIV and four-fold higher in participants with TB and TB/HIV compared to healthy controls (p = 0.0001) (Fig 2). Among the patient groups, the highest expression was in participants with TB/HIV compared to TB or HIV patients alone, although this did not reach statistical significance. In the same figure, PDL1 expression was also assessed on each of three monocytes subsets based on the recent classification [13], and each of the three subsets had elevated PDL1 in all diseased individuals, with the exception of classical monocytes in HIV patients. Importantly, PDL1 expression was higher on IM than CM in HC (p = 0.0003), TB (p = 0.0046), HIV (0.0024) and TB/HIV (p = 0.1145). PDL1 expression on IM was slightly higher (not statistically significant) than NCM in HC, HIV and TB/HIV but not TB. In addition, while the majority of tuberculosis patients were bacteriologically confirmed, we assessed whether exclusion of non-confirmed cases would impact any of the statistical significance was reached in the comparisons we made, and that was not the case (data not shown).

### Different correlations level of PDL1 with microbial and clinical indices of TB and HIV

**Fig 3:**
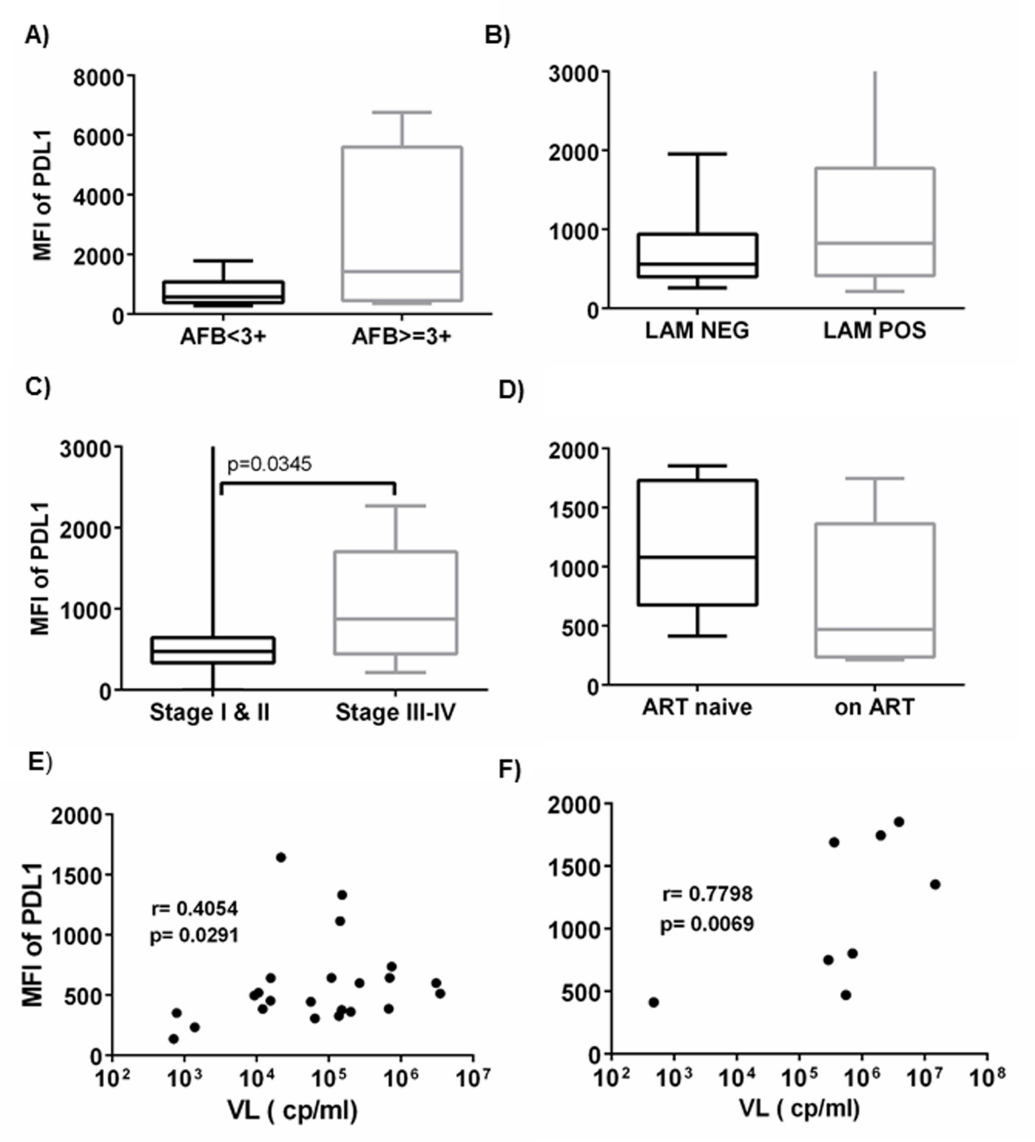
Correlation of microbial derivatives with PDL1. A) PDL1 vs AFB grade in TB and TB/HIV subjects, B) PDL1 vs urinary LAM in TB and TB/HIV, C) PDL1 vs clinical stage of AIDS in HIV and TB/HIV, E) PDL1 vs viral load in HIV, and F) PDL1 vs viral load in TB/HIV. Viral load values were log transformed for data presentation purpose only while statistical analysis was performed on non-transformed data.

Microbial load or their derivatives could have an impact on the expression of PDL1, as they are implicated in continuous stimulation of cells. Fig 3 presents the correlation of the PDL1 with VL, AFB grade and urinary LAM level. None of the mycobacterial indices significantly correlated with PDL1 level expressed on monocytes surface for both participants with TB and TB/HIV. When the participants with TB were categorized into two groups based on their bacillary load (AFB grade above and below 3+), we observed higher median PDL1 levels in the high bacillary load group though the difference was not statistically significant. Similarly, when the subjects were classified into LAM positive and negative, the LAM positive group had higher PDL1 expression but this difference was not statistically significant. On the other hand, there was a significant positive correlation between PDL1 and viral load among HIV positives (r = 0.4054, p = 0.0291) and TB/HIV co-infected cases (r = 0.7798, p = 0.0069). Cases were categorized as mild versus severe disease based on their clinical status, and participants with severe disease had the highest PDL1 expression (p = 0.0345). Furthermore, BMI negatively correlated with PDL1 in all participant groups (r = -0.3616; p = 0.0006). No association between CD4 count and PDL1 was observed.

### Cytokines association with PDL1 in TB, HIV and TB/HIV subjects

**Fig 4:**
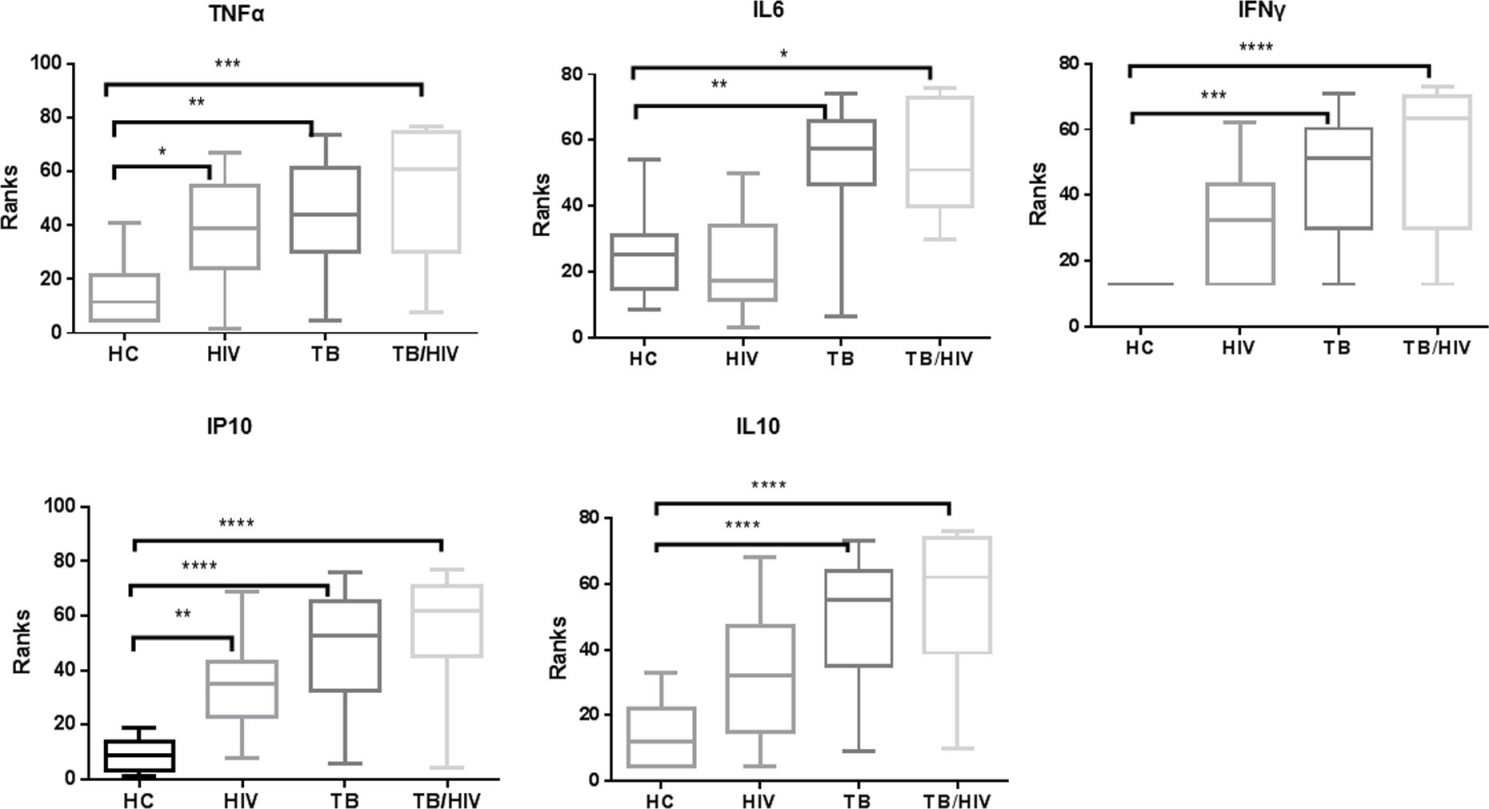
Plasma cytokines level in TB, HIV and TB/HIV subjects. Concentration of TNFα, IL6, IP10, IFNγ and IL10. Group comparison was made by Kruskal-Wallis test followed by Dunn’s multiple comparison test for between groups comparison. The Asterisks represents P-values of: *0.05, **0.01 ***0.001 &****0.0001.

We next addressed whether PDL1 expression on monocytes correlated with serum cytokine levels. Although we assayed 10 cytokine panels (IL1a, IL1β, IFNα, IFNγ, TNFα, IL6, IL10, IP10, IL17, IL12p70), only five of the cytokines (TNFα, IL6, IP10, IFNγ, and IL10) had detectable levels in the majority of subjects. Therefore, the five aforementioned cytokines were included in the analysis. All quantifiable samples have been included in the statistical analysis while zero was substituted for non-quantifiable samples. As described by many previous reports [14], we confirmed the elevation of these cytokines in patients with HIV, TB, and TB/HIV co-infection (Fig 4). The participants with TB/HIV had the highest plasma levels of TNFα, IL6, IP10, IFNγ, and IL10 followed by TB and then HIV participants. Subjects from both TB and HIV cohorts had two-fold higher TNFα than healthy controls whereas TB/HIV patients had five-fold higher TNFα in their plasma. We observed much higher concentrations of IL-6 in the TB and TB/HIV groups, while no differences were seen between HIV and healthy controls for this cytokine. Similarly, significantly high level of IP10 was measured in the plasma of both TB and HIV participants. Though there was increased level of IFNγ in both TB and HIV, but it reached statistical significance only in TB patients. The anti-inflammatory cytokine IL10 concentration was very high in TB/HIV plasma, followed by TB and HIV relative to healthy controls.

**Fig 5:**
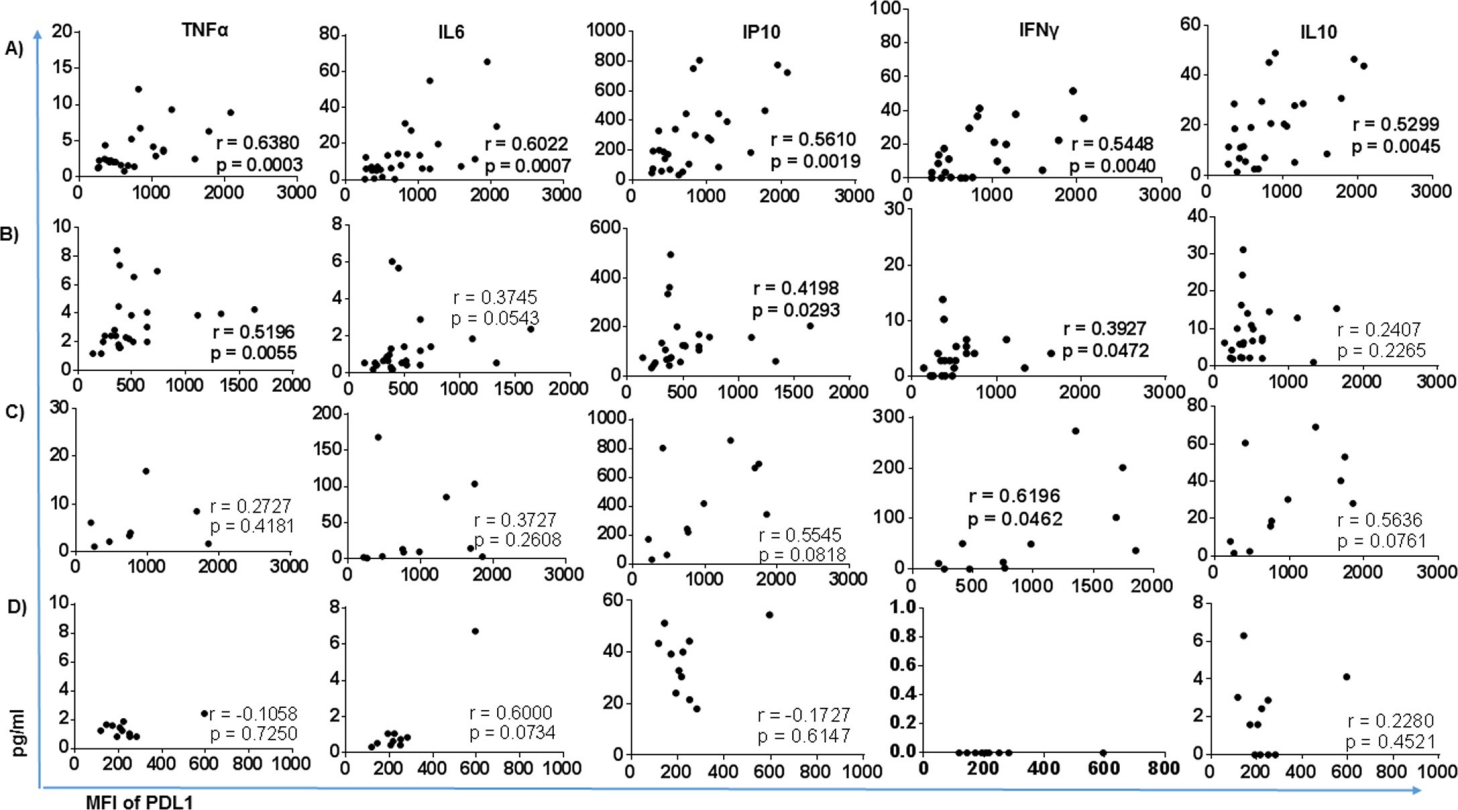
Correlation of PDL1 with plasma cytokine level. The dot-plot represents the correlation of plasma cytokines with PDL1 expression on total monocytes in each study participant group. Correlation was assessed using non-parametric Spearman correlation test and result presented in correlation coefficient(r) along with p-value (p).

The expression of PDL1 on monocytes surface moderately correlated with plasma cytokine levels as presented in Fig 5. PDL1 expression positively correlated with plasma levels of TNFα (r = 0.6280, p = 0.0003), IL6 (r = 0.6022, p = 0.0007), IP10 (r = 0.5610, p = 0.0019), IFNγ (r = 0.5448, p = 0.0040) and IL10 (r = 0.5299, p = 0.0045) in participants with TB. For participants with HIV, PDL1 correlated with TNFα (r = 0.5196, p = 0.0055) and IP10 (r = 0.4198, p = 0.0293) and IFNγ (r = 0.3727, p = 0.0472). PDL1 expression in TB/HIV subjects positively correlated with TNFα, IL6, IP10, IFNγ and IL10, but only that with IFNγ reached statistical significance (r = 0.6196, p = 0.0462).

### Lack of association between cytokine mRNA with PDL1 expression

**Fig 6:**
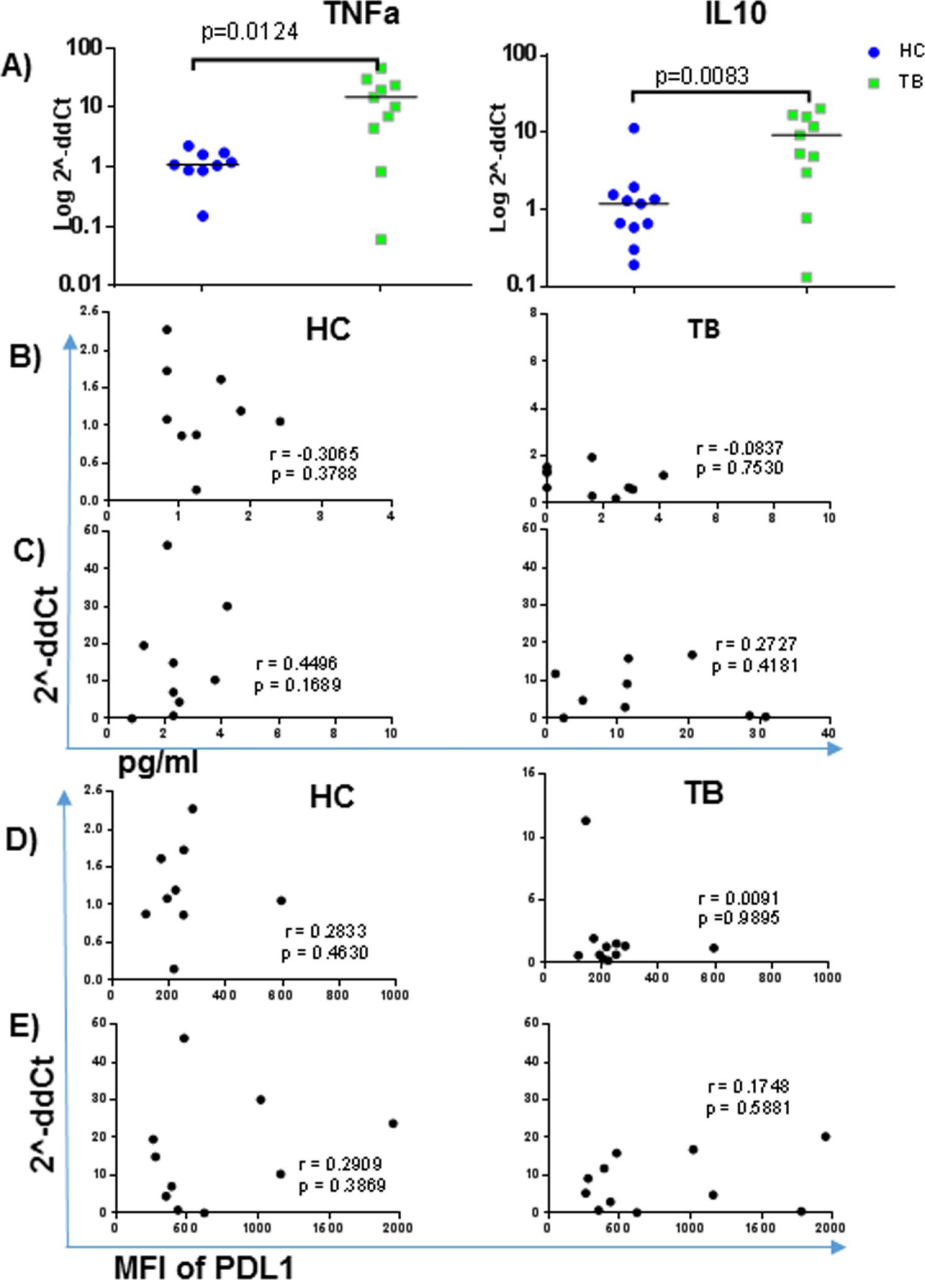
Comparison and correlation of TNFα and IL10 mRNA with plasma cytokine and monocyte PDL1 expression. A) comparison of TNFα and IL10 mRNA expression in TB and HC. The median comparison was calculated by non-parametric Mann Whitney test using 2^-ddCt as comparator. 2^-ddCt values were log-transformed for data presentation purpose only while analysis was done on non-transformed data. B) correlation of TNFα mRNA with plasma cytokines in HC (left) and TB (right), C) correlation of IL10 mRNA with plasma cytokines in HC (left) and TB (right), D) correlation of TNFα mRNA with monocyte PDL1 in HC (left) and TB (right), E) correlation of IL10 mRNA with monocyte PDL1 in HC (left) and TB (right). Expression of surface markers are presented as MFI, mRNA as 2^-ddCt and plasma cytokines in pg/ml.

Given the association between microbes and PDL1, between cytokines and PDL1, and *in-vitro* studies illustrating that microbe induced PDL1 upregulation often involves cytokines, we addressed whether mRNA for cytokines within purified blood monocytes were associated with PDL1 on those monocytes. For this we focused on mRNA levels of TNFα and IL10 from monocytes purified from TB patients (n = 12) and healthy controls (n = 11). Fig 6A demonstrates high levels of cytokine mRNA in TB patients as compared to healthy controls. Despite the significant increase in the cytokine mRNA levels, there was no association between serum cytokine levels and monocyte mRNA level for either TNFα or IL10. Furthermore, there was no association between monocyte PDL1 level with mRNA for these cytokines despite relatively strong correlations of PDL1 with plasma cytokines Fig 6B–6E. Analysis of exclusively confirmed TB cases (representing the majority) did not impact statistical significance of any of the PDL1-cytokine or cytokine mRNA associations.

## Discussion

The purpose of the present investigation was to better define the expression of PDL1 on subsets of human monocytes, and explore the association of expression with clinical states of HIV, TB and TB/HIV co-infection, as well as to assess the correlation of levels with indices of microbial load, serum cytokines and monocyte cytokine mRNA. The key findings are as follows: 1) PDL1 was elevated in monocytes in TB and HIV patients, but importantly, even higher in TB/HIV co-infected patients; 2) although disease augmenting effects were seen in multiple monocyte subsets, the intermediate subset had the highest level of PDL1 and the greatest increase with disease, particularly among TB/HIV co-infected subjects; 3) PDL1 expression was correlated with indices of microbial load in both HIV patients to lesser extent to TB patients, 4) PDL1 expression was correlated moderately with plasma cytokine levels, particularly in TB patients, but not with monocyte cytokine mRNA.

Our observation of elevated expression of PDL1 on monocytes from infected individuals confirms previous studies of TB [15–19], and HIV [7, 20, 21] patients. Importantly, we report the highest level of PDL1 on monocytes from TB/HIV co-infected patients, which to our knowledge have not previously been investigated for PDL1. Given the importance of the PD1 and PDL1 pathway in T cell-antigen presenting cell interactions, this finding further underscores the potential adverse effects of dual infections on immune dysfunction which contribute to worse outcomes in these patients.

There is now a general consensus on the presence of three distinct monocytes subpopulations [13] within human peripheral blood. To our knowledge PDL1 expression has not yet been defined on these subsets, although there have been reports using a previous classification system which delineated CD16+ and CD16- monocytes, but which did not include intermediate monocytes [7]. Our result showed differential expression of PDL1 on the three monocyte subsets both in healthy and patient groups. Although all subsets expressed PDL1, the intermediate monocyte subset (CD16+CD14+) exhibited the highest expression, irrespective of disease condition. While many studies have indicated the different monocyte subsets have a degree of plasticity in terms of their final functional status, it is notable that apart from elevated HLA-DR, the intermediate monocyte subsets have highest levels of other gene transcripts relevant to antigen presentation [22]. Given this apparent tendency towards antigen presenting function, enhanced PDL1 on this subset would be consistent with a potentially greater impact on interacting T cells. Further research into human monocyte subset phenotype and function as well as their interaction with other cells is needed.

We confirm here previous findings of associations of HIV viral load with PDL1 expression [7, 21]. Among multiple mechanisms suggested to underlie this association, the possibility of a direct effect of HIV virion or products upon interaction with monocytes has found support in *in-vitro* studies of normal monocyte PDL1 modulation upon stimulation with HIV tat as well as HIV associated TLR 7,8 agonists [21].

The theoretical possibility that monocyte surface PDL1 is related to TB bacillary load has been strengthened by observations such as reversal of PDL1 modulation with TB therapy, findings of significantly higher levels of PDL1 on pleural fluid derived monocytes as opposed to blood monocytes in patients with pleural TB [16], and experiments demonstrating modulation by intact mycobacteria on monocytes *in-vitro* [18]. However, a convenient surrogate for TB mycobacterial load does not yet exist. We utilized TB smear grade and urinary LAM as potential proxies, and although we did observe positive associations with PDL1 these did not reach statistical significance. In particular, urinary LAM reflects mycobacillary product dissemination into blood, and is hence theoretically attractive, but is limited by sensitivity and expression primarily in TB/HIV co-infected patients. In that the latter cohort was relatively small in our study, future studies with much larger sample sizes, or undertaken with novel markers of TB load. may be needed to substantiate this finding.

In addition to microbes or their products, cytokines have been—implicated in the modulation of PDL1 on human monocytes, most studies employing *in-vitro* approaches. A number of cytokines including IL1β, TNFα, IL-6, IL10, IL12 have shown the ability to induce PDL1 *in-vitro* on human monocytes [7, 17, 23]. We confirmed that many of these cytokines are present in abundance in diseased individuals, as reported elsewhere [14, 24, 25], and further demonstrated significant correlations with PDL1 in our TB, HIV and TB/HIV co-infected patients. Similarly, positive correlations of PDL1 with cytokines such as TNFα and IL-6 have been reported in other chronic inflammatory diseases including systemic lupus erythematous and cancer [23, 26].

Given that blood monocytes are capable of producing these cytokines in response to microbes or their products, and our findings of associations between monocyte PDL1 and many serum cytokines, a reasonable hypothesis would be that peripheral blood monocytes produce and respond to microbial products to release cytokines which then induce PDL1 on these cells. In fact, *in-vitro* PDL1 induction on monocytes has been shown to be at least partly dependent upon cytokines such as TNFα produced in response to microbe stimuli [7].

We therefore assessed the mRNA levels for some of these cytokines (TNFα, IL-10) from purified monocytes, focusing on TB patients and controls. We observed neither a correlation of mRNA with serum cytokines, nor an association between monocyte cytokine mRNA and PDL1. These results imply that—at least in TB patients—if such cytokines are playing a role in modulating PDL1 in peripheral blood monocytes, it is likely not due local production by such monocytes, but rather production from other cellular sources. These could include monocytes or other cytokine producing cells from peripheral tissues at the site of microbial infection or perhaps from cells from other lymphoid organs such as spleen, known to harbor substantial monocyte reserves [27]. More research is needed to better define the source and role of cytokines in modulation of monocyte PDL1.

The main limitation of this study was the lower number of TB/HIV co-infected patients enrolled. This was related to our aim of including patients prior to therapy; more could have been otherwise recruited. We reported a number of associations, but several did not reach statistical significance, likely related to the lower sample size. We opted to raise attention to these associations because of the paucity of monocyte literature on TB/HIV co-infected patients, but clearly these observations should be addressed in future studies with larger samples sizes.

## Conclusion

In the present study we have confirmed previous studies defining the association of PDL1 on monocytes in HIV and TB patients as well as its association with serum cytokines. We have extended these studies and demonstrate for the first time a) elevated levels of PDL1 on monocytes from TB/HIV co-infected patients, b) that among monocyte subsets the highest expression of PDL1 occurs on intermediate monocytes among HIV or TB/HIV co-infected patients, and c) while serum cytokines associated with PDL1, neither serum cytokines nor monocyte PDL1 surface expression associated with blood monocyte cytokine mRNA. These results underscore the importance of further characterization of multiple monocyte subsets and their phenotypic and functional differences in different disease states.

## References

[1] WHO. Global Tuberculosis Report https://wwwwhoint/publications/i/item/9789240013131 2020.

[2] Parrish NM, Dick JD, Bishai WR. Mechanisms of latency in Mycobacterium tuberculosis. Trends in microbiology. 1998;6(3):107–12. doi: 10.1016/s0966-842x(98)01216-5 9582936

[3] Raftery GSaMJ. The PD-1/PD-L1 Axis and Virus Infections: A Delicate Balance. Front Cell Infect Microbiol 9:207. 2019. doi: 10.3389/fcimb.2019.00207 31263684 PMC6584848

[4] Jil M. Jubel ZRB, Christof Burger, Dieter C. Wirtz and Schildberg Frank A. The Role of PD-1 in Acute and Chronic Infection. Front Immunol 11:487. 2020. doi: 10.3389/fimmu.2020.00487 32265932 PMC7105608

[5] Rao M, Valentini D, Dodoo E, Zumla A, Maeurer M. Anti-PD-1/PD-L1 therapy for infectious diseases: learning from the cancer paradigm. International Journal of Infectious Diseases. 2017;56:221–8. doi: 10.1016/j.ijid.2017.01.028 28163164

[6] Day CL, Abrahams DA, Bunjun R, Stone L, de Kock M, Walzl G, et al. PD-1 expression on Mycobacterium tuberculosis-specific CD4 T cells is associated with bacterial load in human tuberculosis. Frontiers in immunology. 2018;9:1995. doi: 10.3389/fimmu.2018.01995 30233588 PMC6127207

[7] Said EA, Dupuy FP, Trautmann L, Zhang Y, Shi Y, El-Far M, et al. Programmed death-1–induced interleukin-10 production by monocytes impairs CD4+ T cell activation during HIV infection Nature medicine. 2010;16(4):452. doi: 10.1038/nm.2106 20208540 PMC4229134

[8] Racquel Domingo-Gonzalez OP, Andrea Cooper, and Shabaana Khader. Cytokines and Chemokines in Mycobacterium tuberculosis infection. 2016. doi: 10.1128/microbiolspec.TBTB2-0018-2016 27763255 PMC5205539

[9] Chong Sun RMaTNS. Regulation and Function of the PD-L1 Checkpoint. Immunity 2018;48. doi: 10.1016/j.immuni.2018.03.014 29562194 PMC7116507

[10] Tara Sassé JW, Li Zhou and Nitin K. Saksena. Monocytes and their Role in Human Immunodeficiency Virus Pathogenesis. American Journal of Infectious Diseases. 2012;8 (2): 92–105.

[11] Livak KJ, Schmittgen TD. Analysis of relative gene expression data using real-time quantitative PCR and the 2− ΔΔCT method. methods. 2001;25(4):402–8. doi: 10.1006/meth.2001.1262 11846609

[12] Mukaka MM. A guide to appropriate use of correlation coefficient in medical research. Malawi medical journal. 2012;24(3):69–71. 23638278 PMC3576830

[13] Loems Ziegler-Heitbrock PA, Suzanne Crowe, Marc Dalod, Veronika Grau, Derek N. Hart, Pieter J., et al. Lutz21. Nomenclature of monocytes and dendritic cells in blood. Blood 2010. doi: 10.1182/blood-2010-02-258558 20628149

[14] Markos Abebe AM. Cytokines and Chemokines as Biomarkers of Tuberculosis. Mycobacterial Diseases. 2013;03(02). doi: 10.4172/2161-1068.1000128

[15] Shen L, Gao Y, Liu Y, Zhang B, Liu Q, Wu J, et al. PD-1/PD-L pathway inhibits M. tb-specific CD4+ T-cell functions and phagocytosis of macrophages in active tuberculosis. Scientific reports. 2016;6:38362. doi: 10.1038/srep38362 27924827 PMC5141449

[16] Pan X, Zhong A, Xing Y, Shi M, Qian B, Zhou T, et al. Increased soluble and membrane-bound PD-L1 contributes to immune regulation and disease progression in patients with tuberculous pleural effusion. Experimental and therapeutic medicine. 2016;12(4):2161–8. doi: 10.3892/etm.2016.3611 27698705 PMC5038224

[17] Emmanuel Stephen-Victor VKS, Mrinmoy Das, Anupama Karnam, Chaitrali Saha, Maxime Lecerf, Caroline Galeotti, et al. IL-1β, But not Programed Death-1 and Programed Death ligand Pathway, is critical for the human Th17 response to Mycobacterium tuberculosis. 2016. doi: 10.3389/fimmu.2016.00465 27867382 PMC5095489

[18] Amar Singh AM, Aparajit B. Dey, and Dipendra K. Mitra. Inhibiting the Programmed Death 1 Pathway Rescues Mycobacterium tuberculosis–Specific Interferon γ–Producing T Cells From Apoptosis in Patients With Pulmonary Tuberculosis. 2013. doi: 10.1093/infdis/jit206 23661793

[19] Syeda S. Hassan MA, Elizabeth C. King, Hazel M. Dockrell, Jacqueline M. Cliff. PD-1, PD-L1 and PD-L2 Gene Expression on TCells and Natural Killer Cells Declines in Conjunction with a Reduction in PD-1 Protein during the Intensive Phase of Tuberculosis Treatment. 2015. doi: 10.1371/10.1371/journal.pone.0137646 PMC4567315 26359860

[20] Day CL, Kaufmann DE, Kiepiela P, Brown JA, Moodley ES, Reddy S, et al. PD-1 expression on HIV-specific T cells is associated with T-cell exhaustion and disease progression. Nature. 2006;443:350. doi: 10.1038/nature05115 https://www.nature.com/articles/nature05115#supplementary-information. 16921384

[21] Meier A, Bagchi A, Sidhu HK, Alter G, Suscovich TJ, Kavanagh DG, et al. Up-regulation of PD-L1 on monocytes and dendritic cells by HIV-1 derived TLR ligands. AIDS (London, England). 2008;22(5):655. doi: 10.1097/QAD.0b013e3282f4de23 18317010 PMC2810187

[22] Wong KLWWHYSMOJJYTTMDSC. The three human monocyte subsets- implications for health and disease 2012. doi: 10.1007/s12026-012-8297-3 22430559

[23] Ou J-N, Wiedeman AE, Stevens AM. TNF-α and TGF-β counter-regulate PD-L1 expression on monocytes in systemic lupus erythematosus. Scientific reports. 2012;2:295. doi: 10.1038/srep00295 22389764 PMC3291882

[24] Adane Mihret MA, Yonas Bekele, Abraham Aseffa, Gerhard Walzl and Rawleigh Howe. Impact of HIV co-infection on plasma level of cytokines and chemokines of pulmonary tuberculosis patients. BMC Infectious Diseases 2014;14:125. doi: 10.1186/1471-2334-14-125 24592945 PMC3974017

[25] Oskar OLSSON PB, Marianne JANSSON, Taye Tolera BALCHA, Daba MULLETA, Habtamu YEBA, Christine VALFRIDSSON, et al. Plasma profiles of inflammatory markers associated with active tuberculosis in antiretroviral therapy naïve HIV-positive individuals. 2015. doi: 10.1093/ofid/ofz015/5304594 PMC6379652 30800697

[26] Hartley G, Regan D, Guth A, Dow S. Regulation of PD-L1 expression on murine tumor-associated monocytes and macrophages by locally produced TNF-alpha. Cancer Immunol Immunother. 2017;66(4):523–35. doi: 10.1007/s00262-017-1955-5 ; PubMed Central PMCID: PMC6469500. 28184968 PMC6469500

[27] Swirski FK, Nahrendorf M, Etzrodt M, Wildgruber M, Cortez-Retamozo V, Panizzi P, et al. Identification of splenic reservoir monocytes and their deployment to inflammatory sites. Science. 2009;325(5940):612–6. doi: 10.1126/science.1175202 19644120 PMC2803111

